# Contrast Agent Pooling (C.A.P.) sign and imminent cardiac arrest: a retrospective study

**DOI:** 10.1186/s12873-022-00634-4

**Published:** 2022-05-06

**Authors:** Yu-Hsuan Lee, Jiashan Chen, Po-An Chen, Jen-Tang Sun, Bo-Hwi Kang, Sheng-En Chu, Chieh-Min Fan, Kuang-Chau Tsai, Shyh-Shyong Sim

**Affiliations:** Department of Emergency Medicine, Far Eastern Memorial Hospital, No. 21, Sec. 2, Nanya S. Rd., Banciao Dist., New Taipei City, 220 Taiwan

**Keywords:** Emergency medicine, Radiology, Shock, Cardiac failure, Resuscitation

## Abstract

**Background:**

The sign of contrast agent pooling (C.A.P.) in dependent part of the venous system were reported in some case reports, which happened in the patients before sudden cardiac arrest. Until now, there is no solid evidence enough to address the importance of the sign. This study aimed to assess the accuracy of the C.A.P. sign in predicting imminent cardiac arrest and the association of the C.A.P. sign with patient’s survival.

**Methods:**

This is a retrospective cohort study. The study included all patients who visited the emergency department, who received contrast computed tomography (CT) scan and then experienced cardiac arrest at the emergency department (from January 1, 2016 to December 31, 2018). We evaluated the occurrence of the C.A.P. sign on the chest or abdominal CT scan, patients with ECMO were excluded. With positive C.A.P. sign, the primary outcome is whether in-hospital cardiac arrest happens within an hour; the accuracy of C.A.P. sign was calculated. The secondary outcome is survival to discharge.

**Results:**

In the study, 128 patients were included. 8.6% (*N* = 11) patients had positive C.A.P. sign and 91.4% (*N* = 117) patients did not. The accuracy of C.A.P. sign in predicting cardiac arrest within 1 h was 85.94%. The C.A.P. sign had a positive association with IHCA within 1 h after the CT scan (adjusted odds ratio 7.35, 95% confidence interval [CI] 1.27 – 42.69). The relative risk (RR) of survival to discharge was 0.90 with positive C.A.P. sign (95% CI 0.85 – 0.96).

**Conclusions:**

The C.A.P. sign can be considered as an alarm for imminent cardiac arrest and poor prognosis. The patients with positive C.A.P. sign were more likely to experience imminent cardiac arrest; in contrast, less likely to survive.

**Trial registration:**

IRB No.108107-E.

**Supplementary Information:**

The online version contains supplementary material available at 10.1186/s12873-022-00634-4.

## Introduction

**Fig. 1:**
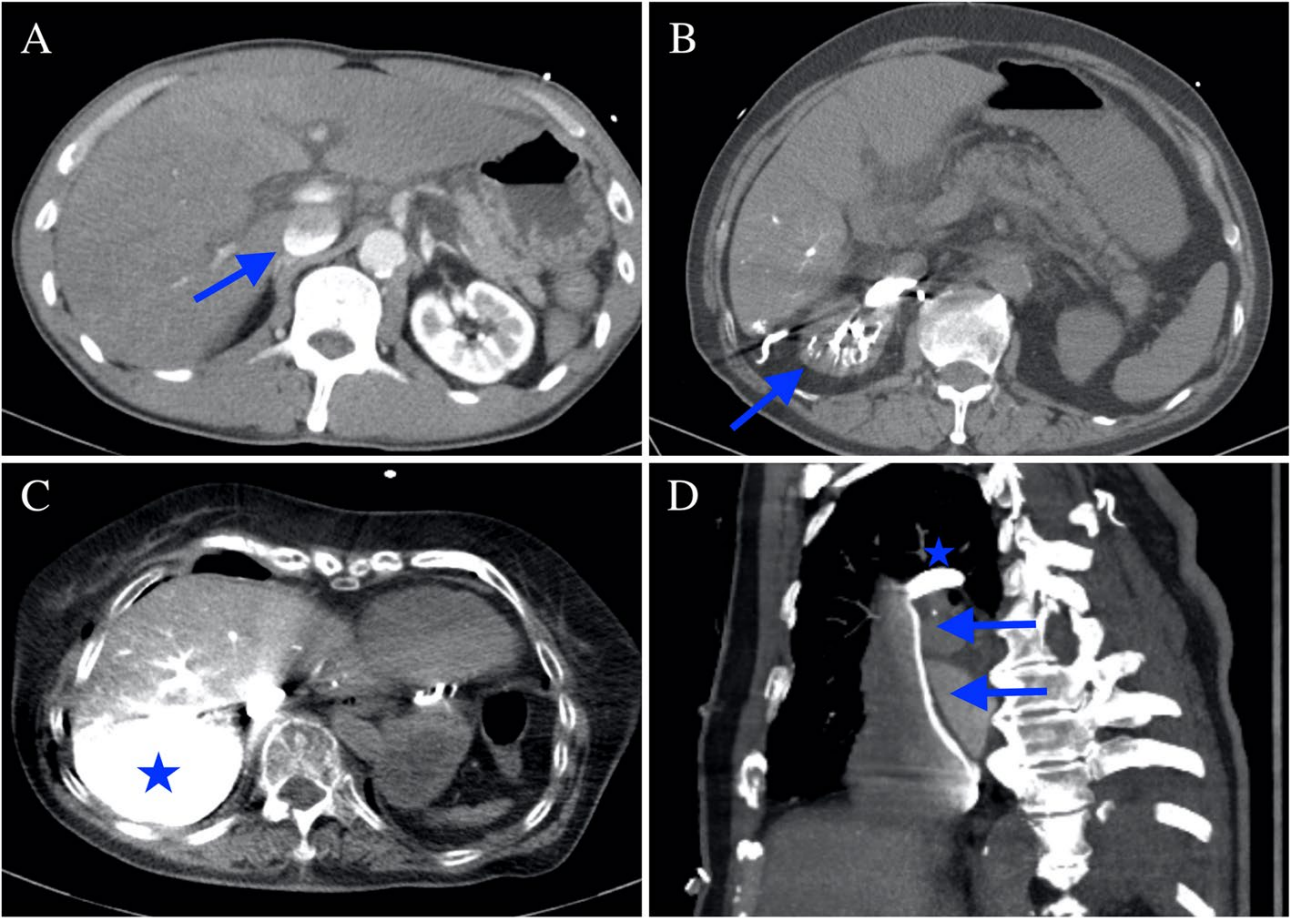
**A** Contrast agent pooling and layering over the inferior vena cava (arrow). **B** Contrast agent pooling over the right renal vein (arrow). **C** Retrograde pooling of the contrast agent over the dependent part of the hepatic veins and parenchyma (star). **D** Contrast agent layering over the superior vena cava (arrows) and pooling over the arch of the azygos vein (star)

In-hospital cardiac arrest (IHCA) remains a largely unpredictable event, which can happen at any time, and has outcomes that are highly dependent on rapid diagnosis and treatment. Multiple studies suggest that healthcare providers often fail to detect changes or abnormalities in the vital signs of patients, hours before an IHCA event [1]. Currently, there are no evidence-based modalities or examinations for predicting cardiac arrest events. Computed tomography (CT) plays an important role in diagnosing diseases. Dependent venous pooling of CT contrast agent was first described in 2002 [2]. CT features of impending cardiac arrest are characterised by pooling of contrast agent in the dependent parts of the right side of the body, including the venous system, right renal vein, and right lobe of the liver (Fig. 1).

This sign was observed in patients with diverse conditions, including dissecting aorta [2, 3], hypovolemic shock caused by trauma accident or internal bleeding [2–5], pulmonary embolism [3, 6, 7], myocardial infarction [8], constrictive pericarditis [8], bilateral pleural effusion and pericardial effusion [9], brain haemorrhage [8], and septic shock [2]. However, C.A.P. sign has only been described in case reports or series.

To the best of our knowledge, this is the first study to evaluate the accuracy of the C.A.P. sign in predicting imminent cardiac arrest.

We hypothesise that the risk of occurrence of cardiac arrest, within 1 h, increases from the detection of the C.A.P. sign on the CT scan. Hence, the C.A.P. sign can be considered a new early warning sign to provide in-time interventions for critically ill patients, thereby preventing cardiac arrest incidents.

## Materials and methods

### Study design and setting

This retrospective cohort study was conducted in an emergency department (ED) of a medical centre between 1 January 2016 and 31 December 2018. The study has been approved by the Research Ethics Review Committee (IRB No.108107-E), which is organized under, and operates in accordance with, the Good Clinical Practice guidelines and government laws and regulations. Informed consent is waived as the study involved retrospective charts and images review. The study adheres to STARD statement whenever possible. The medical centre has 1,300 inpatient beds, has 120,000 yearly visits in the ED, and is the only medical centre in the region with a population of 1,205,570 people.

### Selection of participants

The study included all patients who visited the ED during the study period, underwent intravenous contrast computed tomography (CT), and then experienced cardiac arrest in the ED. Patients who received extracorporeal membrane oxygenation (ECMO) support before CT scan were excluded from the study.

We collected patients’ demographic characteristics (age and sex), underlying comorbidities (hypertension diabetes mellitus, chronic kidney disease, coronary artery disease, heart failure and malignant diseases), the latest vital signs before the CT scan (heart rate and systolic blood pressure), and clinical manifestations (use of inotropic agents and endotracheal intubation). Medians and ranges of age, heart rate, systolic blood pressure were obtained. Shock index was calculated by dividing heart rate by systolic blood pressure. Optiray® 320 ioversol injection 68% was used as an intravenous contrast agent in our hospital. Its specific gravity is 1.371; the average specific gravity of adult blood is approximately 1.0506 at 37 °C [10]. The results of CT scans were evaluated by two senior emergency physicians (with Cohen’s kappa coefficient 0.82) who were masked to C.A.P sign status and patients’ outcomes.

### Measurements

This study evaluates the occurrence of the C.A.P. sign on the chest or abdominal CT scan. It was defined as positive either when the contrast agent accumulated in the renal or hepatic vein or dependent part of liver, or when the contrast agent layering over vena cava was detected.

### Outcomes

The primary outcome was the accuracy of the C.A.P. sign in predicting imminent cardiac arrest (denoted as cardiac arrest occurring within 1 h after the CT scan). The secondary outcome was whether a patient survived up to his or her discharge from the hospital (denoted as survival to discharge) with the presence of C.A.P. sign.

### Statistical analysis

Sensitivity, specificity, positive predictive value (PPV), and negative predictive value (NPV) were calculated by standard formulas for a binominal proportion. The corresponding 95% confidence intervals (CIs) were calculated by the Wilson interval method. The accuracy of the test was determined. The distributions of the selected covariates were compared by the presence of C.A.P. sign. Categorical variables were reported as percentages and analysed with Pearson’s chi-squared tests. A multivariable regression model was established to evaluate C.A.P sign as a predictive factor for primary outcome. Additional variables that showed univariable association with the primary outcome was included in the model if evidence of a significance had been confirmed (*p* < 0.05). A two-tailed *p*-value < 0.05 was considered to be statistically significant. All statistical analyses were conducted using R software, version 3.5.3.

## Results

### Characteristics of the study subjects

In the study period, 726 patients experienced IHCA during their stay in the ED of FEMH. Among them, 138 patients underwent CT before cardiac arrest. Ten patients who received ECMO therapy before the CT scan were excluded, and therefore, 128 patients were included in the study**.**

### Main results

**Fig. 2:**
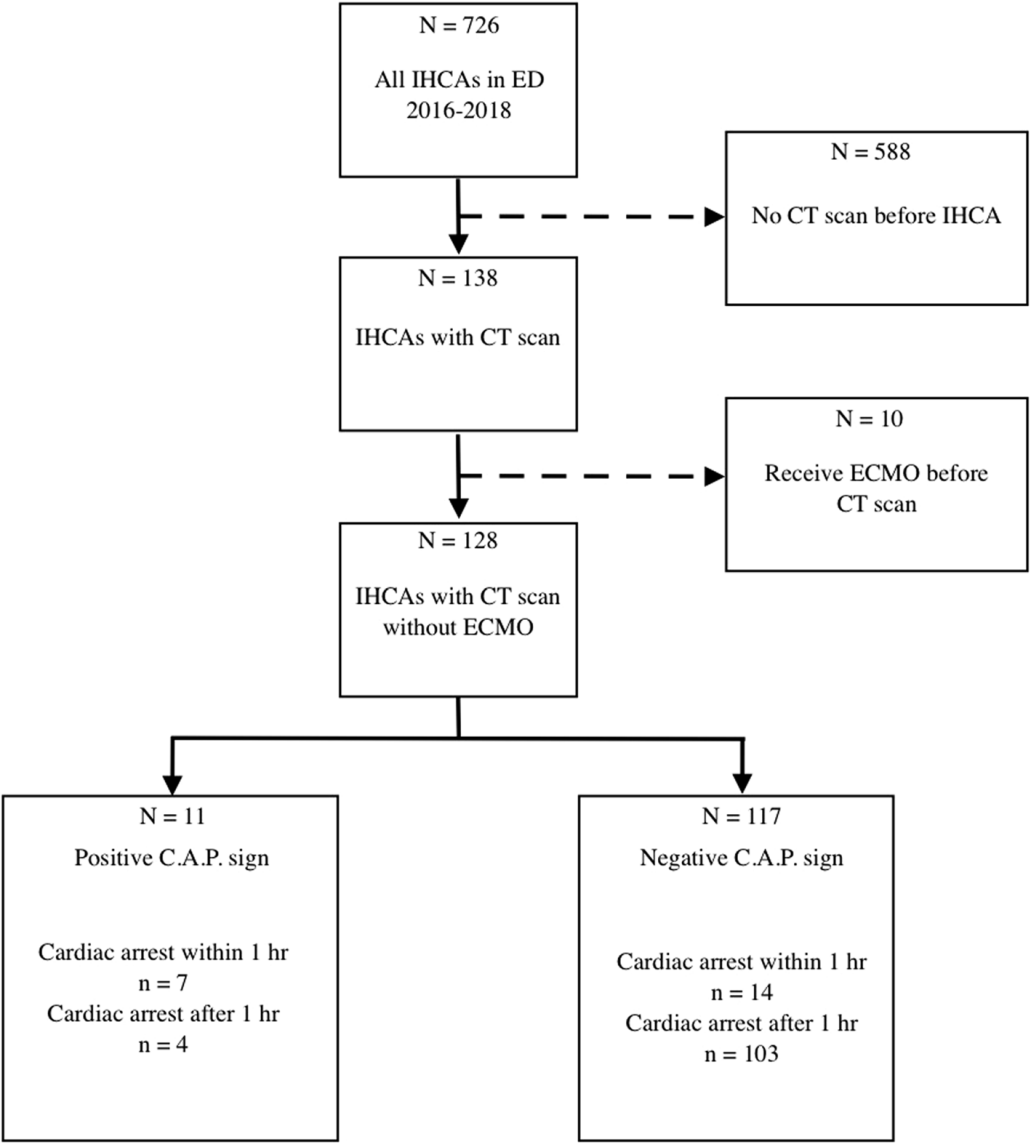
The flow chart of the study population

The CT reports of 8.6% (*N* = 11) of the patients were positive for the C.A.P. sign, whereas the CT reports of 91.4% (*N* = 117) of the patients were negative for the sign (Fig. 2). The overall accuracy of C.A.P. sign in predicting cardiac arrest was 85.94% (95% CI: 78.69–91.45%). The PPV for the C.A.P sign was 64% (95% CI: 36–85%), and the NPV was 88% (95% CI: 84–91%), with 33% (95% CI: 15–57%) sensitivity and 96% (95% CI: 91–99%) specificity.

Demographic characteristics of the study population and outcomes are summarised in Table 1. Most patients were male (60.2%) and were older than 65 years (57%). The profiles of underlying diseases were as follows: history of heart failure (8.6%, *N* = 11), history of coronary artery disease (11.7%, *N* = 15), history of chronic kidney disease (8.6%, *N* = 11), history of diabetes mellitus (22.7%, *N* = 29), history of hypertension (44.5%, *N* = 57), and history of malignant diseases (17.2%, *N* = 22). As for the underlying diseases, there was no significant difference between 2 groups. Before cardiac arrest, heart rates higher than 100 bpm and systolic blood pressure lower than 90 mmHg were detected in 43.8% (*N* = 56) and 39.1% (*N* = 50) of the included patients, respectively. A total of 14.1% (*N* = 18) of the included patients survived to discharge. No patient with a positive C.A.P. sign was diagnosed with diabetes mellitus previously. The heart rates of patients in the group with a negative C.A.P. sign were higher than those of patients in the group with a positive C.A.P. sign (46.2% vs. 18.2%); although, the difference was not statistically significant. Shock index was higher in patients with positive C.A.P sign (1.05 vs 0.85), with most of patients in the group had shock index higher or equal to 0.9 (85.7% vs 43.2%, *p* = 0.03). Regarding to clinical outcomes, patients with positive C.A.P sign had lower survival rate (0% vs 15.4%, *p* = 0.16), and are more likely to experience cardiac arrest within 1 h after the CT scan (63.6% vs 12%, *p* < 0.0001).

**Table 1:** Demographic and clinical characteristics of study population

	**All**	**C.A.P. Sign positive**	**C.A.P. Sign negative**	***p* value**
	(*N* = 128)	(*N* = 11)	(*N* = 117)	
**Demographic**				
Age	69 (21–97)	66 (50–85)	69 (21–97)	0.957
Male sex	77 (60.2%)	8 (72.7%)	69 (59.0%)	0.373
**Clinical**				
Heart failure	11 (8.6%)	1 (9.1%)	10 (8.5%)	0.950
Coronary artery disease	15 (11.7%)	1 (9.1%)	14 (12.0%)	0.776
Chronic kidney disease	11 (8.6%)	1 (9.1%)	10 (8.5%)	0.950
Diabetes mellitus	29 (22.7%)	0 (0.0%)	29 (24.8%)	0.060
Hypertension	57 (44.5%)	3 (27.3%)	54 (46.2%)	0.228
Malignancy	22 (17.2%)	1 (9.1%)	21 (17.9%)	0.456
Heart rate (bpm)	102 (40–192)	87 (50–132)	104 (40–192)	0.172
Heart rate ≧ 100 bpm	56 (43.8%)	2 (18.2%)	54 (46.2%)	0.074
Systolic blood pressure (mmHg)	115 (53–209)	92 (65–132)	118 (53–209)	0.117
Systolic blood pressure < 90 mmHg	50 (39.1%)	7 (63.3%)	43 (36.8%)	0.081
Shock index	0.86 (0.27–2.16)	1.05 (0.38–1.42)	0.85 (0.27–2.16)	0.111
Shock index ≧ 0.9	47 (46.1%)	6 (85.7%)	41 (43.2%)	0.029
Endotracheal intubation before CT scans	67 (52.3%)	7 (63.6%)	60 (51.3%)	0.433
Inotropic agent usage before CT scans	42 (32.8%)	4 (36.4%)	38 (32.5%)	0.793
**Outcomes**				
Cardia arrest within 1 h after CT scans	21 (16.4%)	7 (63.6%)	14 (12.0%)	< 0.0001
Survival to discharge	18 (14.1%)	0 (0%)	18 (15.4%)	0.160

Data are recorded as N (%) or median (range)

*CT* Computed tomography

Table 2 shows the odd ratios and their 95% CIs for selected variables. Among those, cardiac arrest within 1 h after CT scans was significantly associated with positive C.A.P sign (OR 12.88, 95% CI 3.34 to 46.634). Shock index ≧ 0.9 was slightly associated with positive C.A.P sign (OR 7.90, 95% CI 2.92 to 68.22).

**Table 2:** Univariate analysis of factors associated with positive C.A.P. sign

	**Odd Ratio**	**95% Confidence Interval**	**p value**
Age ≧ 65	1.35	0.38—4.87	0.64
Male sex	1.86	0.47—7.35	0.38
Heart failure	1.07	0.12—4.87	0.95
Coronary artery disease	0.74	0.09—6.19	0.78
Chronic kidney disease	1.07	0.12—9.24	0.95
Hypertension	0.44	0.11—1.73	0.24
Malignancy	0.46	0.06—3.77	0.47
Heart rate ≧ 100 bpm	3.86	0.8—18.63	0.09
Systolic blood pressure < 90 mmHg	0.33	0.09—1.2	0.09
Shock index ≧ 0.9	7.90	2.92—68.22	0.03
Endotracheal intubation before CT scans	1.66	0.46—5.98	0.44
Inotropic agent usage before CT scans	1.19	0.32—4.31	0.79
Cardiac arrest within 1 h after CT scans	12.88	3.34—49.63	< 0.001

The C.A.P sign was positively associated with episode of cardiac arrest within 1 h after CT scans (aOR 7.35, 95% CI 1.27 to 42.69) and negatively associated with survival to discharge (RR 0.9, 95% CI 0.85 to 0.96). Likewise, shock index ≧ 0.9 could be identified as a predictor factor for both clinical outcomes (aOR 1.70, 95% CI 0.47 to 6.07; RR 0.33, 95% CI 0.10 to 1.12), though it was not statistically significant. The results are presented in Table 3.

**Table 3:** Predictive factors for clinical outcomes

Cardiac arrest within 1 h after CT scans			
	Adjusted* Odd Ratio	**95% Confidence Interval**	***P* value**
C.A.P. sign	7.35	1.27 – 42.69	0.026
Shock index ≧ 0.9	1.70	0.47 – 6.07	0.417
*adjusted for C.A.P. sign and shock index			
**Survival to Discharge**			
	**Relative Risk**	**95% Confidence Interval**	
C.A.P. sign	0.90	0.85 – 0.96	
Shock index ≧ 0.9	0.33	0.10 – 1.12	

The clinical and imaging findings, as well as the outcome of the patients with positive C.A.P. sign were summarized in Table 4. Among 11 patients, there were 10 patients (90.9%) with contrast agent pooling at IVC, 6 patients (54.5%) with contrast agent pooling at hepatic veins, 2 patients (18.2%) at renal veins and 1 patient (9.1%) at SVC. No patient in this group was discharged alive.

**Table 4:** The clinical and imaging findings of the patients with positive contrast agent pooling sign

Case	Gender	Age	Diagnosis	CT Findings	Timing of Cardiac arrest (after CT scan)	Survival to Discharge
1	F	66	Lymphoma with leukostasis	Contrast agent layering in hepatic vein; poor perfusion of spleen and kidneys	1-h 40-min	N
2	M	76	Cardiac tamponade, pneumonia	Contrast agent layering in IVC; massive pericardial effusion; bilateral lung consolidations and pleural effusion	5-h 15-min	N
3	M	81	Abdominal aortic aneurysm, rupture	Contrast agent pooling in IVC and renal veins; infrarenal abdominal aortic aneurysm rupture with moderate hemoperitoneum	immediate	N
4	F	85	Ribs fracture with hemopneumothorax, pelvic fracture	Contrast agent pooling in IVC and hepatic veins; multiple ribs fracture with effusion; pelvic fracture with retroperitoneal bleeding	7-min	N
5	M	66	Spleen laceration with internal bleeding	Contrast agent pooling in IVC and hepatic veins; massive hemoperitoneum	53-min	N
6	M	77	Traumatic subarachnoid hemorrhage, subdural hematoma, lung contusion	Contrast agent pooling and layering in IVC; ground glass opacities over bilateral lungs	8-h	N
7	M	85	Aortic dissection with cardiac tamponade	Contrast agent pooling in IVC, right renal veins and right dependent part of liver and hepatic veins; Contrast agent layering in SVC; type A aortic dissection with hemopericardium	12-min	N
8	M	59	Acute myocardial infarction	Contrast agent layering in IVC; poor contrast enhancement of left ventricular wall	25-min	N
9	M	63	Acute myocardial infarction	Contrast agent pooling in IVC; poor contrast enhancement of left ventricular wall	12-min	N
10	M	50	Severe metabolic acidosis	Contrast agent pooling and layering in IVC; contrast agent pooling in hepatic veins	56-min	N
11	F	57	Corrosive injury of upper gastrointestinal tract	Contrast agent pooling in IVC and hepatic veins; extensive wall swelling of esophagus, stomach, duodenum with poor contrast enhancement	1-h 20-min	N

*IVC* Inferior vena cava, *SVC* Superior vena cava

**Fig. 3:**
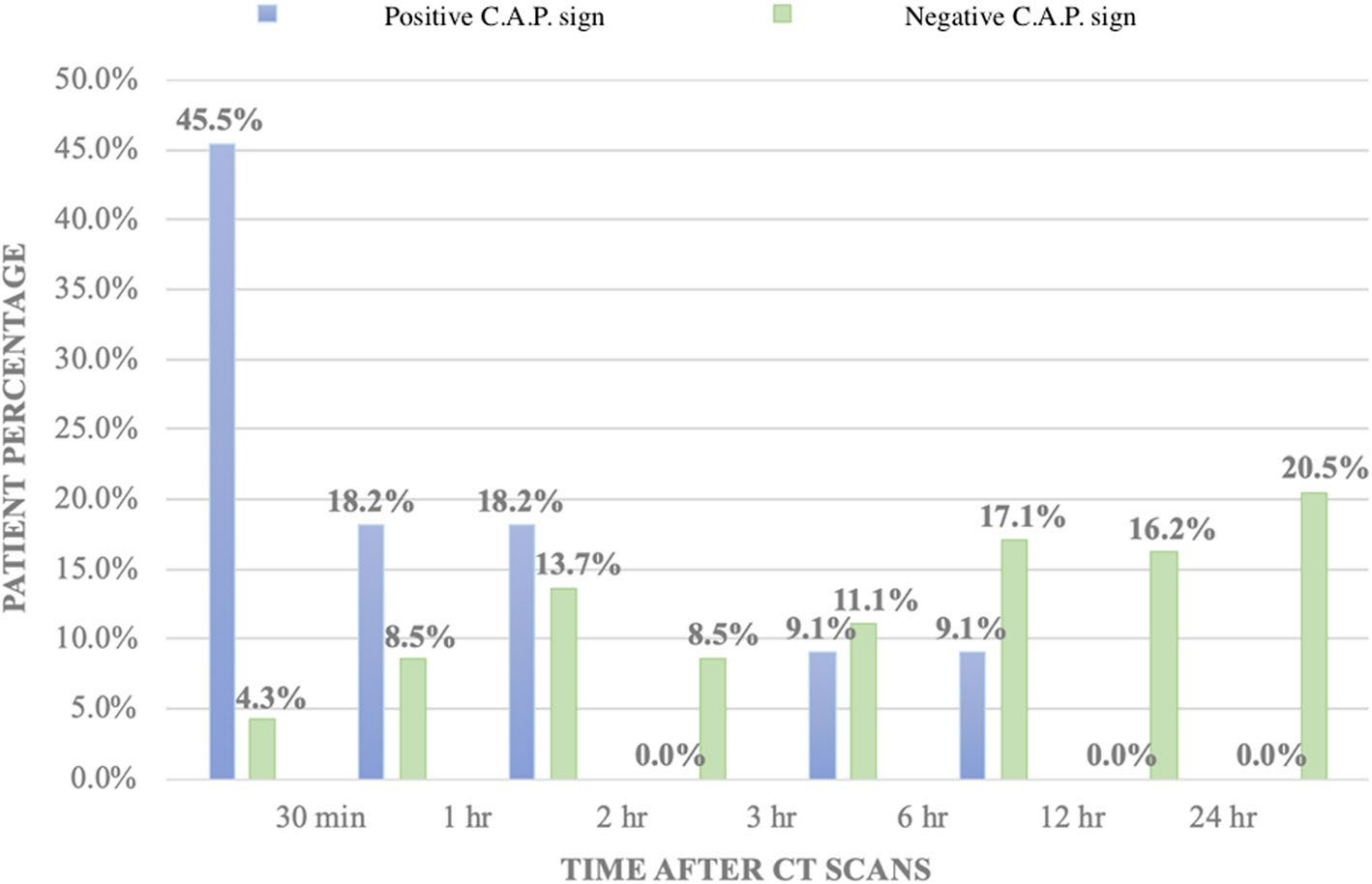
Timing of cardiac arrest after the CT scan

Changes in IHCA incidence percentage with respect to time are presented in Fig. 3. About 45.5% of the patients with a positive C.A.P. sign collapsed within 30 min after the CT scan. A total of 7 out of the 11 patients (63.7%) with a positive C.A.P. sign collapsed within 1 h after the CT scan. In contrast, only 4.3% of the patients with a negative C.A.P. sign were reported as having IHCA within 30 min after the CT scan, and the incidence percentages increased with time.

## Discussion

The results of this study reveal that the presence of the C.A.P. sign on the CT scan strongly associated with the likelihood of imminent cardiac arrest. The patients with positive C.A.P. sign were more likely to experience cardiac arrest within 1 h after the CT scan. In our study, all the patients with positive C.A.P. sign failed to survive; our result also showed that the C.A.P. sign negatively associated with survival to discharge slightly.

C.A.P. sign occurs owing to the effect of gravity. The density of contrast agent is much higher than that of blood; thus, during normal physiological blood flow, specific gravity has little effect on the contrast agent dynamics. In patients with cardiogenic shock or impending circulatory collapse, both the arterial and venous blood flow dramatically decreases, and the contrast agent tends to accumulate in the dependent parts of the venous system [11]. Additionally, a lack of sufficient blood pressure during shock also inhibits penetration into the organ parenchyma and contributes to the pooling of contrast agent in major vessels [8]. About 52% of the cases  demonstrate this effect of gravity after cardiac arrest [12].

In a retrospective study that reviewed 127 patients who underwent contrast-enhanced CT of the chest or abdomen and echocardiography, high injection rate (> 3 ml/sec), tricuspid regurgitation, pulmonary hypertension, and right ventricular systolic dysfunction were found to be an independent predictor of retrograde contrast agent pooling over inferior vena cava or hepatic vein (*p*< 0.001, < 0.01, = 0.05, and < 0.005, respectively) [13]. This study further demonstrated that the phenomenon of contrast agent pooling is caused by weak venous blood flow and may be influenced by contrast agent injection rate.

We found 25 articles, reporting 59 cases with the sign of dependent contrast agent pooling [2–29]. Age ranges from 6 to 87-year-old. 36 of 59 are male, 21 are female and 2 patients without mentioning of gender. The CT scans with contrast of chest and abdomen were arranged for the survey of trauma (18 cases), aortic dissection, pulmonary embolism, sepsis and abdominal mass, as shown in the supplementary table [Additional file 1].

The site of contrast agent pooling is mostly found over inferior vena cava (IVC). Contrast agent pooling at IVC was described in 55 cases (93.2%). In 4 cases without contrast agent pooling at IVC, 3 cases are traumatic patients with massive hemoperitoneum, one of them with ruptured diaphragm and herniation of liver, the increased intraperitoneal pressure may suppress contrast agent regurgitation in IVC. In second patient without contrast agent pooling was with Type A aortic dissection, contrast agent refluxed at hepatic and lumbar vein. Besides of IVC, contrast agent pooling was also observed in sporadic cases over right hepatic vein, left hepatic vein, dependent liver parenchyma, right renal parenchyma, lumbar vein, splenic vein, superior mesenteric vein, superior vena cava, right atrium, right ventricle, coronary sinus, great cardiac vein, azygos vein and hemiazygos vein.

Thirty-nine of fifty-nine cases (66.1%) were reported to experience cardiac arrest within 30 min after CT. 35 of 39 cases developed cardiac arrest immediately after CT. Among 59 cases with contrast agent pooling sign, there were 10 cases (16.9%) with cardiac tamponade, 5 cases (8.5%) with cardiogenic shock. In 54 cases which mentioned about clinical outcome in literature, 44 cases (81.5%) expired eventually.

There was one case (1.7%) who did not experience cardiogenic shock or cardiac arrest despite of positive contrast agent pooling sign; however, extreme low ejection fraction was noted at follow up echocardiography [20].

Four pediatric cases were found in literature review, three of them were collapsed soon after CT scan [17, 29]. One case with history of chronic constrictive pericarditis developed neither cardiac arrest nor cardiogenic shock was proven with severe right ventricular failure at follow up echocardiography.

Summarized from literature reviews and our cases, the feature of C.A.P. sign includes contrast agent pooling in IVC, right hepatic vein, left hepatic vein, dependent liver parenchyma, right renal parenchyma, lumbar vein, splenic vein, superior mesenteric vein, superior vena cava, right atrium, right ventricle, coronary sinus, great cardiac vein, azygos vein and hemiazygos vein. The most common site of contrast agent pooling is IVC (93.2% from the reported cases, 90.9% from our case series).

In our study, 63.7% of the patients with a positive C.A.P. sign experienced cardiac arrest within 1 h, and all patients with a positive C.A.P. sign experienced cardiac arrest within 8 h. Our result coincides with those of the previous studies, indicating that C.A.P. sign may be used as a predictor for imminent cardiac arrest.

Contrast-enhanced computed tomography may not be a diagnostic tool for impending cardiac arrest and severe cardiogenic shock. Nevertheless, if the patients underwent CT to survey critical illnesses, the physician has an opportunity to inspect for the C.A.P. sign. Recognition of the sign can effortlessly alert primary care physicians, and rapidly indicate the possibility of extremely low cardiac output or impending circulatory failure. Hence, C.A.P. sign should grab physician’s attention to activate timely evaluation and earlier intervention to save the patient’s life. In some cases, earlier termination of CT may be necessary to provide immediate cardiopulmonary resuscitation and prevent end-organ ischemia or death. In clinical scenario, if a patient with positive C.A.P. sign is clinically unstable, we propose earlier intubation and inotropic agent usage to stabilize the patient, and rapidly search and treat potential reversible causes.

This study has several limitations. First, the presentation of dependent venous pooling is not well defined. In some cases, contrast agent pooling was noted to be confined to the right hepatic lobe, right renal vein, inferior or superior vena cava, and even subclavian vein. This condition may imply specific haemodynamic disturbances in these cases.

Second, there are no statistical data on the incidence rate of C.A.P. sign in the normal population or the population with low cardiac output. Further studies are needed to investigate the incidence rate of C.A.P. sign in the normal population and in populations with extremely low cardiac output. Measurement of cardiac output during contrast-enhanced CT may provide more information about the development of the sign.

Third, selection bias. Our study focuses on the CT findings of the patients experienced cardiac arrest in emergency department of single medical centre. The statistic values of C.A.P. sign may not apply to all population. However, as the occurrence of C.A.P. sign in normal population is extremely rare, we believe the accuracy of C.A.P. sign in predicting cardiac arrest would be much higher if the study could include more patients.

## Conclusions

From our findings, the C.A.P. sign could be addressed as an imaging feature of circulatory failure, it could be used to predict imminent cardiac arrest and should be considered a warning sign for clinical physicians to provide in-time interventions for critically ill patients.

## Supplementary Information

**Additional file1.**

## Data Availability

The datasets used and/or analyzed during the current study are attached as a related file [deID_CAPs_raw.xlsx] during submission.
